# Genomic-enabled classification of three *Mycobacteroides abscessus* subspecies and an effective subspecies-specific identification method

**DOI:** 10.1128/jcm.00697-25

**Published:** 2025-07-24

**Authors:** Zelin Yu, Ruibai Wang

**Affiliations:** 1 National Key Laboratory of Intelligent Tracking and Forecasting for Infectious Disease, National Institute for Communicable Disease Control and Prevention, Chinese Centre for Disease Control and Prevention 96698, Beijing, Beijing, China; The University of North Carolina at Chapel Hill School of Medicine, Chapel Hill, North Carolina, USA

**Keywords:** *Mycobacteroides abscessus*, average nucleotide identity, core gene, *erm*(41), subspecies, identification

## Abstract

*Mycobacteroides abscessus* (Mab) is a clinically significant non-tuberculous mycobacterium. It comprises three distinct subspecies being considered to have different macrolide susceptibilities, transmission patterns, and treatment outcomes. In this study, systematic analysis was conducted on 2,006 Mab genomes deposited in the National Center for Biotechnology Information genome database, and the taxonomic classification of their subspecies was revised accordingly. The findings revealed that: (i) in terms of three distinct subspecies classification, the analysis based on core genes and average nucleotide identity (ANI) values was completely consistent; (ii) ANI was a reliable criterion for Mab species and subspecies classification, with defined thresholds of 95% ANI for species-level and 98% ANI for subspecies-level differentiations; and (iii) the integrity of the *erm*(41) gene or the similarity of the *rpoB* gene was an unreliable characteristic for Mab subspecies, and the assertions that subspecies *massiliense* lack inducible resistance to macrolides also cannot be sustained. Moreover, through a subspecies re-classification of genomes and pangenome analysis, Mab subspecies-specific genes were successfully identified, and a novel single-gene test with enhanced clinical applicability was developed. Additionally, the impact of reference genome selection on taxonomic classification highlighted the importance of adopting a standardized set of reference genomes in species/subspecies identification to significantly enhance the comparability across different studies.

**IMPORTANCE:**

*Mycobacteroides abscessus* (Mab) is a clinically challenging non-tuberculous mycobacteria species. The accurate identification of subspecies is of utmost importance for clinical diagnosis and treatment, as well as for research on pathogenicity, drug resistance, and other related aspects. This study provided a clear average nucleotide identity threshold for Mab subspecies classification, as well as revised options of the three Mab subspecies, new and accurate Mab subspecies-special biomarker, and a detection technique with practical clinical application.

## INTRODUCTION

*Mycobacterioides abscessus* (homotypic synonym: *Mycobacterium abscessus*, Mab) is an important pathogenic non-tuberculous mycobacteria (NTM) species that can cause respiratory, skin, and mucosal infections in humans. Among these infections, Mab pulmonary infections are the most challenging of all NTM infections for a relatively low average treatment success rate (1) partially owing to their intrinsic resistance to traditional anti-tuberculosis drugs (2). Mab is the most prevalent NTM species in China (3) and ranks only second to the *Mycobacterium avium* complex in NTM pulmonary infections in the United States (4). Epidemiological evidence suggests an overall increase in Mab infection and disease in the last decade (5).

Mab is a taxonomy puzzle and has undergone several changes (6). It was first isolated in a patient with multiple soft tissue abscesses in 1953 (7). At that time, apart from human, bovine, and avian tuberculosis bacilli and leprosy bacilli, there were few reports on human infections caused by acid-fast bacteria. In 1972, based on the matching matrix generated using 195 characters in a cooperative numerical taxonomic analysis of rapidly growing mycobacteria of Runyon’s group IV conducted by the International Working Group on Mycobacterial Taxonomy, Mab was classified under *Mycobacterium chelonei* (or *M. chelonae*) as a subspecies, though it had sufficient difference from *M. chelonei* subsp. *chelonei* in five tests, including citrate utilization (8). While in 1992, it was precisely due to the differences in biochemical tests and DNA reannealing values (<70%) with *M. chelonae* that Kusunoki and Ezaki demonstrated Mab was a distinct species and proposed to restore its species status (9). However, because the differences between their 16S rDNA sequence were <10 bp and closely related to *M. immunogenum*, they are sometimes referred to as the “*M. chelonae-abscessus* group” (10), an item generally associated with poor prognosis in the literature (11).

In 2004 and 2006, *M. massiliense* and *M. bolletii* were identified, respectively, and classified as new species within the *M. chelonae-abscessus* group based on *rpoB* gene similarity and DNA-DNA hybridization (DDH) (12, 13). In 2009, because of conflicting identification results based on the *rpoB* and *hsp65* sequences, Leao et al. (14) conducted an extensive characterization of the five members of the *M. chelonae-abscessus* group. Their results led to the merger of *M. massiliense* and *M. bolletii* and the set of two subspecies, subsp. *abscessus* and subsp. *massiliense*. The latter was corrected as subsp. *bolletii* in accordance with the Rules of the Bacteriological Code (1990 Revision) in 2011 (15). The study by Bryant et al. based on whole-genome sequencing (WGS) in 2013 (16) and the study by Tortoli et al. in 2016 (17) both divided Mab into three subspecies. In 2017, Adekambi et al. proposed reinstating *M. massiliense* and *M. bolletii* as independent species (18), but this recommendation was not adopted. In 2018, Mab was classified into the genus *Mycobacteroides* with the division of the genus *Mycobacterium* by Gupta et al. (19)

The three Mab subspecies have been reported to have notable differences in several key characteristics. For instance, a treatment success rate of 56.7% was reported for Mab subspecies *massiliense*, whereas it was merely 33.0% for subspecies *abscessus* (20). The sensitivity to macrolides—core drugs in the multi-drug combination regimen for Mab infection—and the related *erm*(41) genotype were found to differ significantly among these subspecies (21). Maurer et al. discovered that the subspecies *abscessus* and *bolletii* possess a full-length functional 522 bp *erm*(41) gene. In contrast, the subspecies *massiliense* harbors a truncated non-functional *erm*(41) gene, measuring only 246 bp (22). This was considered the reason for the inducible macrolide resistance of the subspecies *abscessus* and *bolletii* and the sensitivity of the subspecies *massiliense* to macrolides. Additionally, subspecies *massiliense* was suspected to transmit among cystic fibrosis patients via contaminants or aerosols, while subspecies *abscessus* clustering appeared to result from dominant circulating clones acquired independently from the environment (16, 23). Therefore, clinical Mab isolates needed to be identified accurately to subspecies level, which is crucial for predicting patients' prognoses, summarizing the characteristics of these strains and epidemiological analysis.

The classification methods for mycobacterial species have evolved from Runyon’s phenotypic method (24), numerical taxonomy analysis (25), single- and multi-gene sequencing methods of conserved housekeeping genes, such as 16S rRNA, *rpoB*, and *hsp65*, to the current WGS method. Notably, genetic variations within the *erm*(41) gene among Mab subspecies have also been utilized as the basis for Mab subspecies identification methods (26, 27). However, multi-gene analysis, including multi-locus sequence typing (MLST), has lost its practical utility for two key reasons: the sequencing costs for seven to eight genes now exceed those of second-generation WGS. Meanwhile, the resolution and informational depth of WGS-based analysis far surpass those of multi-gene sequencing.

The average nucleotide identity (ANI) and DDH are measures of the genomic similarity. Accordingly, 95–96% ANI threshold has been used to delineate prokaryotic species, which corresponds to 70% DDH cutoff (28, 29). The Genome-to-Genome Distance Calculator (GGDC) provides digital DDH to replace traditional DDH and recommends 79% as the threshold for subspecies (30, 31). However, there is currently no widely accepted threshold for ANI in defining prokaryotic subspecies, except one study that utilized a 98% ANI criterion to distinguish subspecies within *Salmonella enterica* (32). Tortoli et al. discovered an ANI gap of 97.7–98.4% among 46 Mab strains (17); whether this threshold can be applied to all Mab remains to be verified. With the large number of whole-genome sequences now available, genome-based classification methods can provide more reliable subspecies boundaries. Therefore, in this study, we employed core-gene phylogenetic analysis and large-scale ANI analysis to precisely define the ANI thresholds among the three Mab subspecies and revised the species annotations of all the deposited genomic sequences. However, WGS-based analysis also has its shortcomings. It requires sufficient DNA and at least 24–48 h of testing time, both of which limit the application of this method in routine rapid clinical diagnosis. So subsequently, we identified subspecies-specific regions and transformed ANI-based Mab subspecies identification into a polymerase chain reaction (PCR) assay for two genes to improve the clinical applicability while maintaining the testing accuracy.

## MATERIALS AND METHODS

### Mab genome retrieval and processing

All Mab genomes were obtained from the National Center for Biotechnology Information (NCBI) genome database (https://www.ncbi.nlm.nih.gov/datasets/genome/) before 22 December 2024. To maintain the genome quality, genomes with RefSeq accession were included, and atypical, metagenome-assembled, and suppressed genomes were excluded. Consequently, this study encompassed 2,006 genomes, of which 85 genomes were fully assembled; 979 were annotated as subspecies *abscessus*, 403 as subspecies *massiliense*, and 129 as subspecies *bolletii*. Notably, the remaining 495 genomes lacked subspecies-specific information.

### Phylogenetic, ANI, and DDH analyses of 85 complete genomes

To get a more precise and robust evaluation of ANI’s discriminatory power for Mab subspecies, all 85 fully assembled genomes (Table S1) were analyzed to avoid any potential confounding effects of sequencing and assembly quality on the accuracy and reliability of the results. The genome of *M. chelonae* strain CCUG 47445 (GCF_001632805.1) was selected as the outgroup due to its appropriate phylogenetic distance from Mab, allowing for a clear resolution of intraspecies relationships. The type strains, Mab ATCC 19977 (GCF_000069185.1), subspecies *massiliense* CCUG 48898 = JCM 15300 (GCF_000497265.2), and subspecies *bolletii* BD (GCF_003609715.1) were used as the three subspecies reference genomes.

Prokka v1.14.6 (https://github.com/tseemann/prokka) (33) was used to annotate the genomes. The output GFF files from Prokka were used as input files for Roary v3.13.0 (https://github.com/sanger-pathogens/Roary) (34) to conduct a pan-genome analysis and a multi-FASTA alignment of core genes. The phylogenetic tree was constructed using IQtree v2.3.6 (https://github.com/iqtree/iqtree2) (35) with automatic optimal model selection and 1,000 bootstrap replicates. The *rpoB* genes were extracted from genomes using BLAST v2.16.0+ (http://blast.ncbi.nlm.nih.gov/Blast.cgi), followed by multiple sequence alignment using Muscle v5.3. All trees were visualized and annotated using Tree Visualization By One Table (tvBOT) (36).

ANI comparison was performed using skani v0.2.2 (https://github.com/bluenote-1577/skani) (37) with default parameters. ANI clustering was implemented through a helper Python script provided by skani. Hierarchy function from Scipy v1.13.1 (https://docs.scipy.org/doc/scipy/) was integrated to execute and store hierarchical clustering. The DDH calculation between 85 genomes and three reference genomes was performed using GGDC 3.0 (31). Data analysis and visualization were performed using the tidyverse package (https://www.tidyverse.org/) in R version 4.4.2 (https://www.r-project.org/).

### ANI comparison of all genomes and subspecies threshold evaluation

Pairwise ANI values between 2006 Mab genomes were calculated by skani. Each genome was designated as the reference genome in turn, and all genomes, including the one serving as the reference itself, were set as query genomes. This process resulted in a total of *n*^2^ pairwise comparisons and was converted into ANI matrix. The paired ANI value distribution trends were analyzed and visualized using Gaussian kernel density distribution functions in R.

### Comparison of *erm*(41) gene

Based on the subspecies classification results, we used the gene of Mab ATCC19977 (MAB_RS11715) and a truncated *erm*(41) gene (246 bp) from the subspecies *massiliense* as reference sequences, and extracted the *erm*(41) genes from all 2006 Mab genomes using BLAST v2.16.0+. The detailed procedure was as follows: first, aggregate all genomic files and leverage the makeblastdb command to construct a local database (this consolidation of genomic data creates a comprehensive repository for subsequent analysis); next, utilize the sequence of the target gene as a reference template; and employ the blastn command to conduct a search for homologous sequences within the newly established database. To ensure the precision and comprehensiveness of the search results, specific key parameters should be configured: -outfmt 5 (to generate an output in XML format and facilitate in-depth exploration of the results; -max_hsps 1 (to restrict the display to only the top-scoring pair for each target sequence); and -evalue 1e – 5 (*E*-value threshold to 0.00001). Finally, we developed and executed a Python script to establish a mapping relationship between the sequence ID of each genome and its corresponding genome GCF number and to extract and integrate the homologous sequences of the target gene along with their genomic source information from the XML output file.

### Pangenome analysis and identification of the subspecies-specific genes

After checking the subspecies classification of the 85 complete genomes through ANI and phylogenetic analysis, we used packages, such as Tidyverse in R, to conduct further analysis of the pangenome characteristics of the subspecies on the Rtab file generated by Roary. The Rtab file contains the gene presence/absence matrix for each genome. Under the framework of two key definitions, namely, subspecies-shared and subspecies-specific genes, we used set operation functions, including intersect(), setdiff(), and union(), to analyze the data and delineate the gene distribution patterns among the three Mab subspecies. Subspecies-shared genes are genes that are present in all genomes within one Mab subspecies. However, these genes may also be found in some or all genomes of other two Mab subspecies. In contrast, subspecies-specific genes are unique to one Mab subspecies, being present in all of its genomes while being completely absent from the genomes of the other two Mab subspecies.

### Primer designed and verification using actual strains

Subspecies-specific gene segments for primer design were selected based on pan-genome analysis results, and BLAST v2.16.0+ was used to verify that these differences exist across all 2,006 genomes. Primers were designed using Oligo 6.0 following the general principle of the PCR primer design. Their specificity was verified using Primer-BLAST. Twenty-five strains of the three Mab subspecies and six strains of commonly encountered clinically pathogenic *Mycobacterium* were used for the verification of the identification method. They were 19 strains of the subspecies *abscessus*, five strains of the subspecies *massiliense*, one strain of the subspecies *bolletii* (type strain DSM 45149), two strains of *M. tuberculosis*, and one strain each of *M. avium*, *M. intracellulare*, *M. gordonae*, and *M. kansasii*. All strains were stored in the *Mycobacterium* strain library of the tuberculosis department of the National Institute for Communicable Disease Control and Prevention and were whole-genome sequenced using either second- or third-generation sequencing technologies.

Boiled DNA templates from the fresh cultures of the tested strains were used in PCR. The 50 µL PCR mixture was formulated as follows: 1 µL DNA, 25 µL 2× EasyTaq PCR SuperMix (TransGen Biotech, Beijing, China), and 2 µL 10 µM each primer; the remaining volume was made up to 50 µL with purified water. PCR amplification condition was 5 min at 95°C, followed by 30 cycles of 95°C 30 sec, 58°C 30 s, and 72°C 1 min, with a final extension step at 72°C for 5 min. The PCR products were visualized by 1% agarose gel electrophoresis.

## RESULTS

### Clustering of 85 complete genomes and the ANI boundary of three subspecies

**Fig 1:**
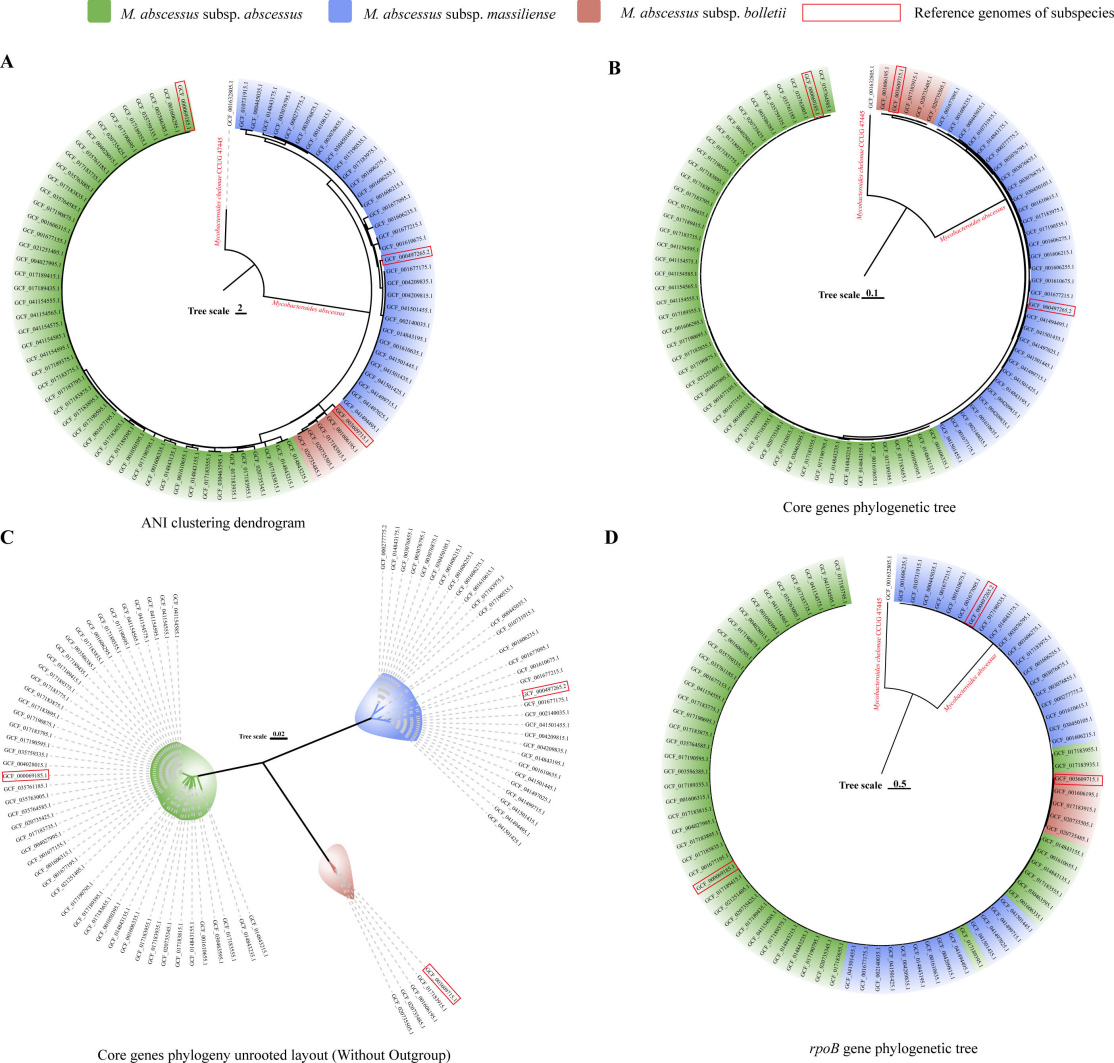
Phylogenetic tree and ANI clustering dendrogram of 85 complete Mab genomes. A, B, and D respectively represent the ANI clustering dendrogram, core gene phylogenetic tree, and *rpoB* gene phylogenetic tree. C represents an unrooted core gene phylogenetic tree without an outgroup.

The ANI clustering effectively segregated all 85 genomes into three distinct clusters. This outcome was fully congruent with the phylogenetic tree constructed based on the core genes (Fig. 1A and B). The topological structure for subspecies is more clearly presented in the unrooted phylogenetic tree without outgroup (Fig. 1C) based on 3,633 core genes identified through the prokka and roary pipeline. However, on the clustering tree constructed based on the *rpoB* gene, which has been commonly used to differentiate the Mab subspecies (6), the genomes indeed could not be classified as well as by the core genes (Fig. 1D).

**Fig 2:**
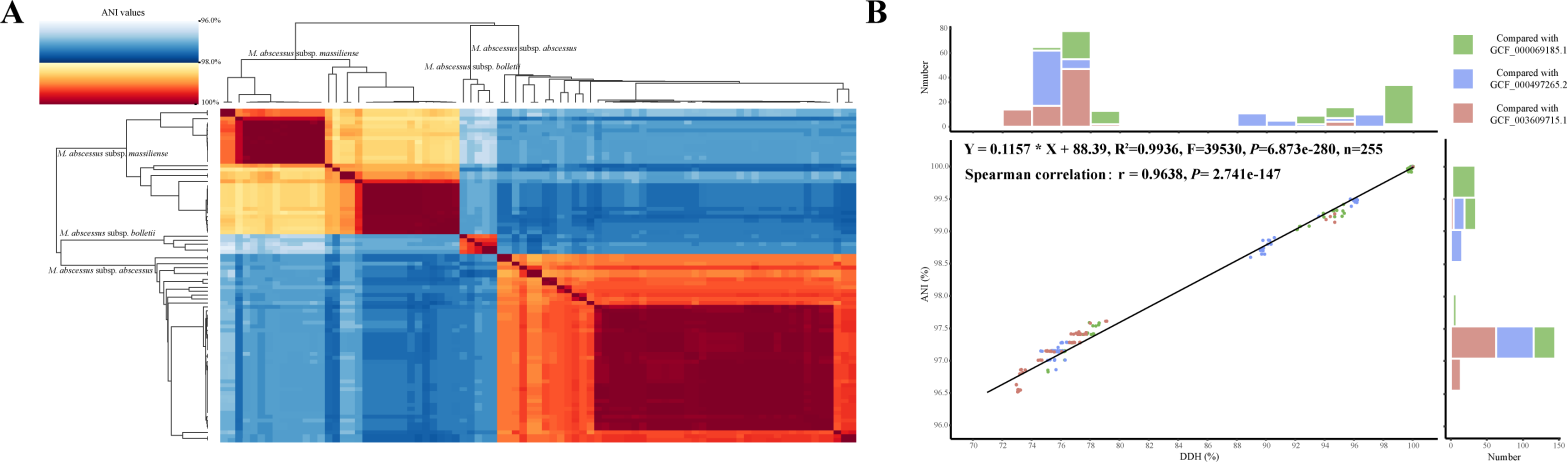
ANI clustering heatmap (**A**) and ANI-DDH scatter plot (**B**). (A) 98% ≥ ANI ≥ 96% are colored in blue, while 100% ≥ ANI > 98% are colored in red. Deeper color intensity corresponds to higher values. (B) ANI and DDH values between all genomes and three subspecies reference genomes. The *X*-axis shows the DDH values, and the *Y*-axis represents the ANI values. Frequency histograms showed the dot numbers. Different colors represent comparisons with different reference genomes.

Notably, distinct ANI boundaries were observed among subspecies (Fig. 2A). Genomes in the same subspecies exhibited ≥98.2% ANI value, while their ANI values with genomes of the other two subspecies were ≤97.7%. There is a good linear regression relationship between the ANI values and their corresponding digital DDH (dDDH) values (*R*^2^ = 0.9936, Fig. 2B). There is a numerical gap with values ranging from 79 to 89% for DDH and 97.75 to 98.5% for ANI both within and across subspecies. The 79% DDH subspecies threshold, as indicated by the best-fitting straight line, corresponds to an ANI value of 97.5%.

### ANI analysis of all genomes and subspecies threshold evaluation

**Fig 3:**
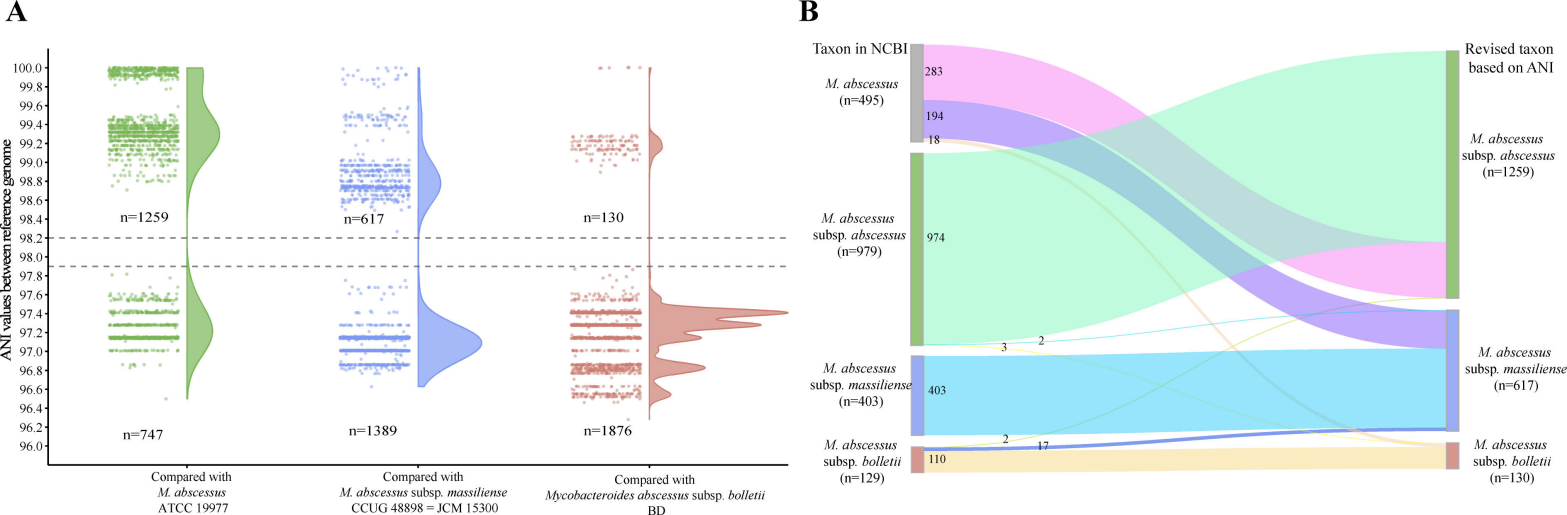
Half violin plot of the ANI value distribution and revised classification. (A) Distribution of the ANI values calculated between all 2,006 genomes and three reference genomes. “N” is the genome numbers above or below the threshold. (B) Sankey diagram of the subspecies classification, with the subspecies annotation in the NCBI database on the left side and the ANI-based revised taxonomy on the right side.

The further ANI comparative analysis was extended to all 2,006 Mab genomes. First, the ANI values between each Mab genome and the three subspecies reference genomes were calculated. The results showed that among the three ANI values of all genomes, one was greater than 98.2%, and the other two were less than 97.9% (Fig. 3A). The ANI values distributed within two regions, namely, greater than 98.2% and less than 97.9%, and there exists a clear subspecies boundary. Based on these findings, we proposed setting 98%, the integer value of the midpoint of the observed range as a clearer and more memorable threshold for Mab subspecies.

Applying this 98% ANI threshold to the reference genome of type strain, a comprehensive taxonomic revision of Mab genomes was conducted. As illustrated in Fig. 3B, all 2,006 Mab genomes could be accurately assigned to one of three subspecies. Specifically, among the initial 979 genomes annotated as subspecies *abscessus*, 974 genomes were confirmed to belong to this subspecies, whereas two genomes were reclassified as subspecies *massiliense* and three as subspecies *bolletii*. All 403 genomes previously classified as subspecies *massiliense* remained without reclassification. Conversely, among the 129 genomes labeled as subspecies *bolletii*, 19 were found by this ANI threshold to be misclassified: two genomes were reclassified as subspecies *abscessus* and 17 as subspecies *massiliense*. A review of their assembly release dates revealed that 14 of the 17 genomes were released between May 2012 and May 2013 (Table S2) (15). This timeline aligns with the taxonomic reclassification period when *M. massiliense* and *M. bolletii* were merged under *M. bolletii*, which elucidates the observed misclassification. For the 495 genomes lacking initial subspecies annotation, taxonomic analysis revealed that 283 belonged to subspecies *abscessus*, 194 to subspecies *massiliense*, and 18 to subspecies *bolletii*. Following these corrections, the final classification comprised 1,259 genomes of subspecies *abscessus*, 617 genomes of subspecies *massiliense*, and 130 genomes of subspecies *bolletii*. Detailed ANI values and classification are listed in the Table S2.

**Fig 4:**
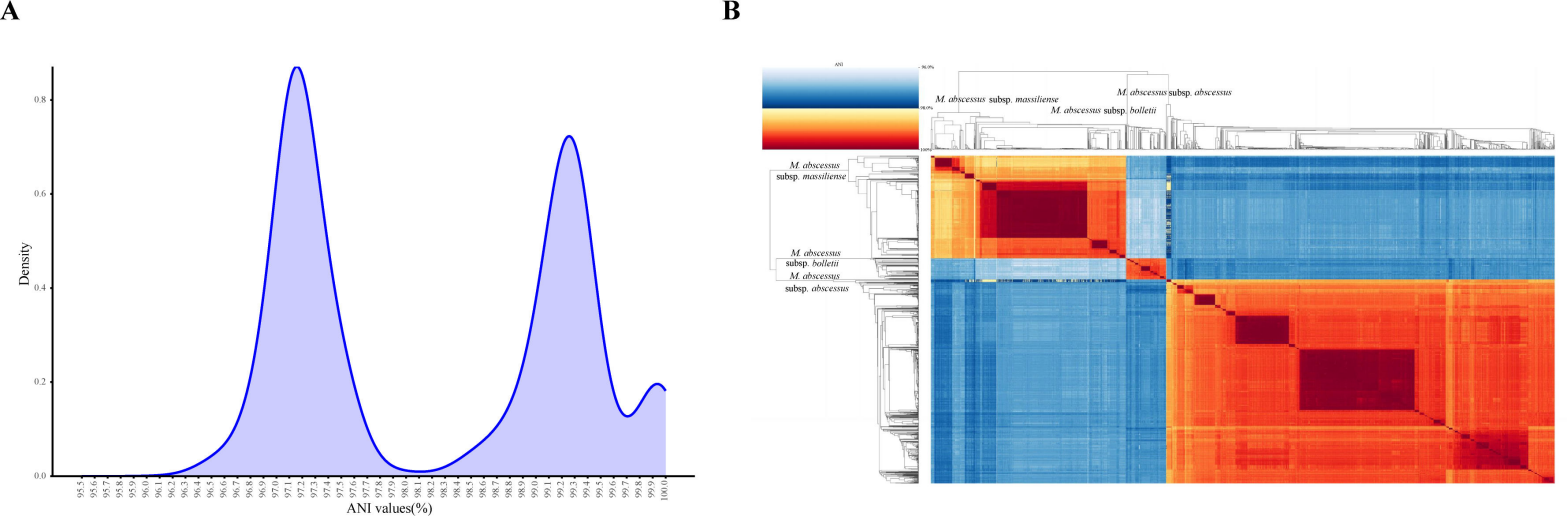
Distribution and clustering of pairwise ANI values. (A) Kernel density plots of the distribution of paired ANI values of all Mab genomes. (B) ANI clustering heatmap of all 2,006 genomes similar to Fig. 2A.

To gain a more comprehensive insight into the population relationships among Mab genomes, pairwise ANI values were further calculated between every two of the 2,006 Mab genomes. In total, 4,024,036 values were output, all of which exceeded 95.86% (Fig. 4A). This finding underscores the notable intraspecific genomic homology and relative conservatism of Mab genomes. From a taxonomic perspective, these data support the utility of a 95% ANI threshold (more precise, 95.8%) for species-level classification of Mab. The distribution of pairwise ANI values manifested a bimodal pattern characterized by a distinct trough positioned just above 98% (Fig. 3A). As the ANI clustering heatmap of 86 complete genomes, all 2,006 genomes could still be clustered into three major clades corresponding to the three subspecies. However, when the analysis was extended to all genomes, the ANI-based subspecies boundaries were less clear than in the previous heatmap (Fig. 4B). Specifically, in contrast to the clear separation, with intra-clade ANI >98.2% and inter-clade ANI ≤97.7%, in pairwise comparison of 86 complete genomes (Fig. 2A), the comparison of 2,006 genomes revealed that a few intra-subspecies ANI values were slightly <98%, while certain inter-subspecies values were higher than this threshold. Frustratingly, adjusting the 98% threshold, whether upward or downward, cannot fully resolve this matter.

**Fig 5:**
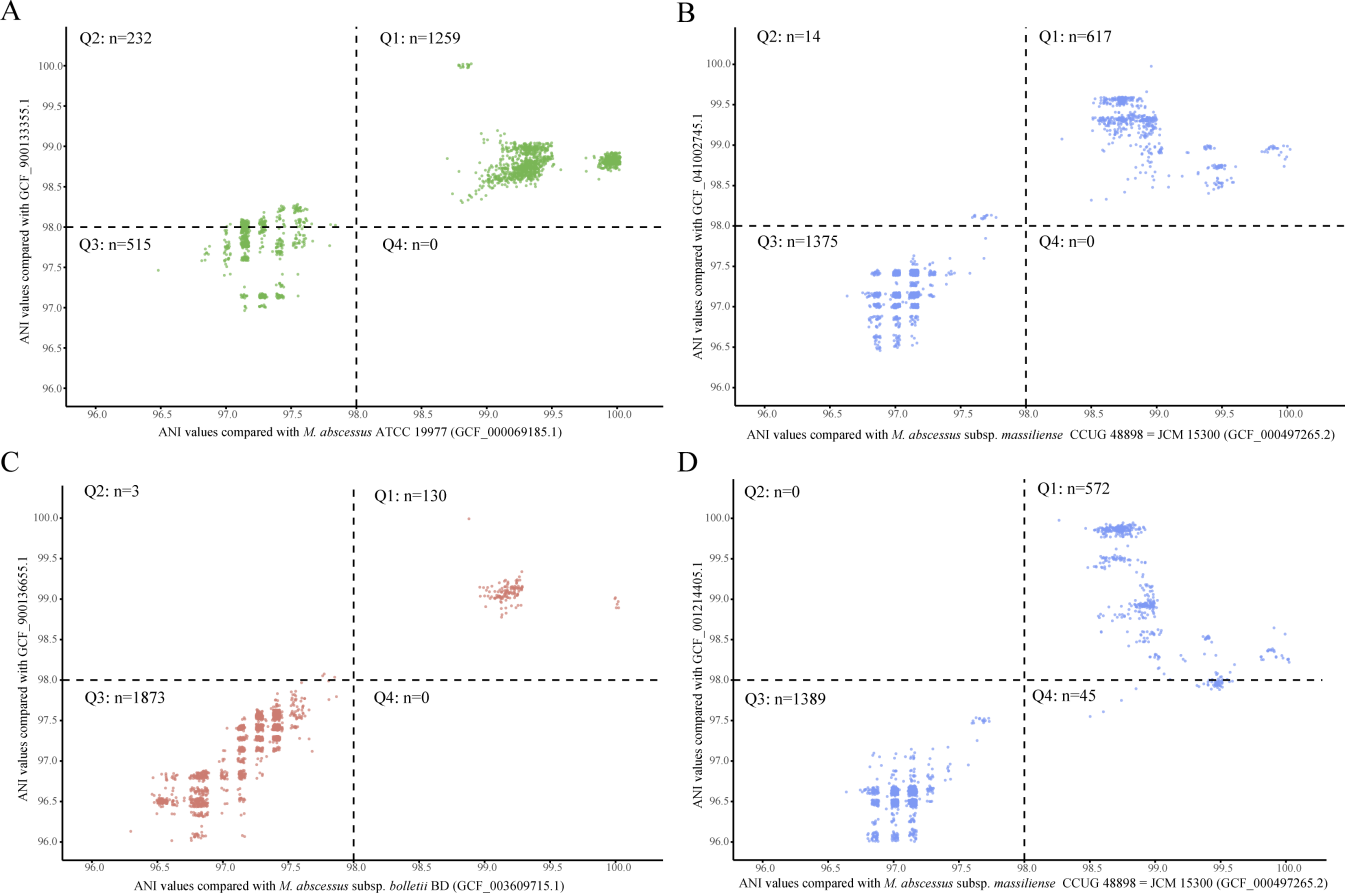
Influence of the reference genome selection on the subspecies classification of Mab genomes presented using a jitter scatter plot. The *X*-axis shows the ANI values compared to the genomes of subspecies type strains, and the *Y*-axis represents the ANI values between genomes and another designated reference genome.

The pairwise ANI values of all Mab genomes also enabled us to recognize the importance of the reference genome selection for the correct classification of species/subspecies. If other genomes in the same subspecies were designated as reference and replaced the type strains' genomes, partial genomes that are determined to be outside the subspecies using the reference genome of the type strain (ANI < 98%) will be included within the subspecies (ANI > 98%) (Fig. 5A through C, the Q2 quadrant). However, when the selected reference genome differs significantly enough from other genomes within the subspecies, an opposite result will also be obtained. As in Fig. 5D, though the ANI of GCF_001214405.1 to the genome of type strain (GCF_000497265.2) was >98%, its ANI values to some genomes within the same subspecies *massiliense* were below 98%. If using GCF_001214405.1 as reference, 45 genomes not belonging to subspecies *massiliense* will be classified in this subspecies.

### Analysis of the key macrolide resistance genes, *erm*(41)

The *erm*(41) gene has a total length of 522 bp. Among 1,259 genomes of subspecies *abscessus*, genomes GCF_003582985.1 and GCF_017175935.1 harbored the 246 bp deletion genotype of *erm*(41) gene, characterized by a 2 bp deletion at positions 64–65 and a continuous 274 bp deletion following position 158. Among 617 subspecies *massiliense* genomes, 605 genomes (98.06%) carried the 246 bp deletion genotype, and one genome (GCF_041002745.1) had a 14 bp insertion within the 246 bp region, resulting in an extended gene length of 260 bp. The remaining 11 genomes (1.78%) harbored intact 522 bp *erm*(41) gene. Additionally, all 130 genomes of subspecies *bolletii* possessed the full-length *erm*(41) gene. Among the two genomes of subspecies *abscessus* with incomplete *erm*(41) genes, one was initially annotated as subspecies *abscessus*, and the other was reclassified from previously unassigned genomes. Among the 11 subspecies *massiliense* genomes with complete *erm*(41) genes, two genomes were originally annotated as subspecies *massiliense*, and nine were reclassified from unassigned genomes. These findings were discrepant with previous conclusions that the presence of intact and functional *erm*(41) genes was a defining genomic feature of subspecies *abscessus*, while defective and non-functional *erm*(41) genes were consistently characteristic of subspecies *massiliense* (1, 22, 27, 38). To ensure the reliability of our findings, we conducted a comprehensive phylogenetic analysis using core genes to re-evaluate these genomes and confirmed the accuracy of their subspecies classification (Fig. S1).

### Identification of Mab subspecies-specific genes

Through pangenome analysis, the commonalities and differences among the three subspecies in 85 complete genomes were presented. All Mab genomes shared 3,633 core genes and 24, 46, and 59 subspecies-specific genes were identified for subspecies *abscessus*, *massiliense*, and *bolletii,* respectively.

**Fig 6:**
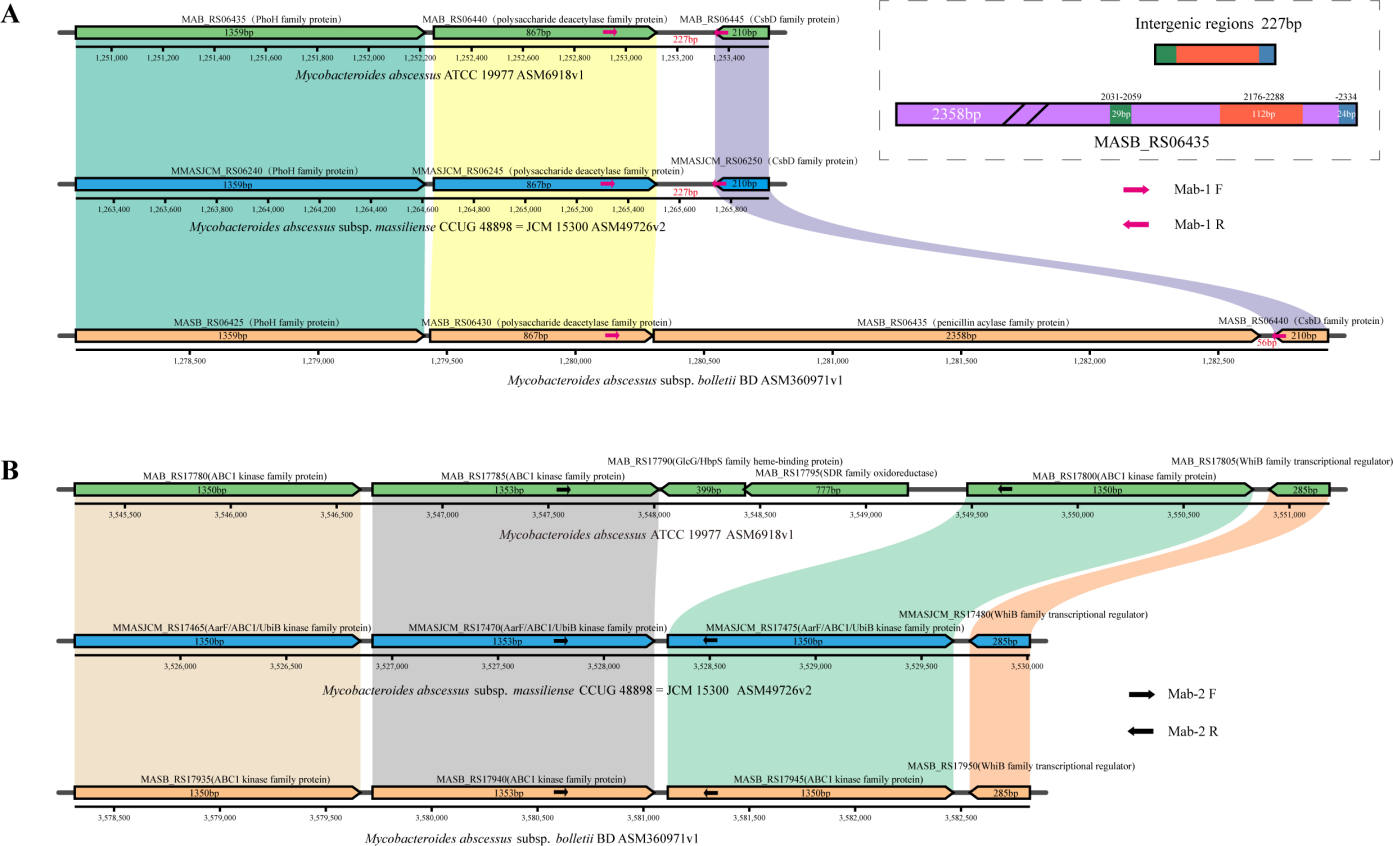
Gene arrangement diagram of two subspecies-specific regions. The positions of the two sets of primers are marked on the map. The upper right corner shows a schematic diagram of the homologous fragment of the MASB_RS06435 gene in subspecies *bolletii* compared to the intergenic regions of other subspecies.

In order to reduce the dependence on equipment and enhance the applicability of the method in basic-level laboratories, our design principles for the identification method of Mab subspecies are not relying on sequencing and the sizes of the products being easily disguised. Finally, two genes, MASB_RS06435 and MAB_RS17790, were selected (Fig. 6).

MASB_RS06435 is a specific gene of subspecies *bolletii*, encoding the penicillin acylase family protein. It is located between MASB_RS06430 and MASB_RS06440, which encode the polysaccharide deacetylase family protein and the CsbD family protein, respectively. In the corresponding regions of subspecies *abscessus* and *massiliense*, the homologous genes of MASB_RS06430 and MASB_RS06440 are directly adjacent. Only a few fragments within the non-coding intergenic region exhibit high similarity to the 3′ end of the MASB_RS06435 gene. In detail, the 29 bp sequence from positions 5 to 33 of the intergenic region is identical to the 29 bp sequence from positions 2031 to 2059 of the MASB_RS06435 gene; the 112 bp sequence from positions 34 to 145 of the intergenic region corresponds to the 113 bp sequence from positions 2176 to 2288 of the MASB_RS06435 gene (with a single base deletion); the sequence after position 146 of the intergenic region is basically the same as the sequence after position 2334 bp of the MASB_RS06435 gene. Based on this, it is judged that these are remains of gene deletion of MASB_RS06435 in subspecies *abscessus* and *massiliense*. This deletion results in the intergenic regions between two genes, MAB_RS06440 and MAB_RS06445, in subspecies *abscessus*, with MMASJCM_RS06245 and MMASJCM_RS06250 in subspecies *massiliense* being only 227 bp in length.

MAB_RS17790 and its downstream gene MAB_RS17795 are specific genes of subspecies *abscessus*, with no matching fragments in the other two subspecies. These two genes encode GlcG/HbpS family heme-binding protein and SDR family oxidoreductase, inserting in the three consecutive coding genes of the ABC1 kinase family protein.

### Mab subspecies detecting method based on PCR

Based on the two regions, two sets of specific primers were designed: Mab-1F (5′-AGT TGA CCG GCA AGT AGT TC-3′) and Mab-1R (5′-CAG CTA TGG CAG CAG AGA G-3′) for PCR1; and Mab-2F (5′-TGC CTC CCG TCT ACC TGA TG-3′) and Mab-2R (5′-CCG CCT TCT CCA AGA GTT CG-3′) for PCR2. Primer-BLAST was used to conduct alignment analysis on the core nucleotide (nt) library of five mycobacteria related genera (*Mycobacterium*, *Mycobacteroides*, *Mycolicibacillus*, *Mycolicibacter*, *Mycolicibacterium*) to detect the specificity of the primers. The results showed that, except for Mab, all genomes, including *M. chelonae* in the same genus of Mab, did not produce amplification products. The primers theoretically have high specificity.

**Fig 7:**
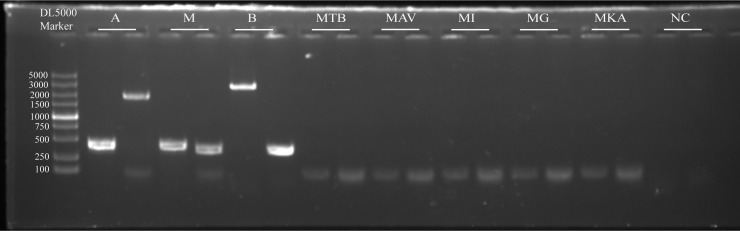
PCR amplification results of actual samples. Mab-1 and Mab-2 refer to two sets of primers; A, M, and B represent subspecies *abscessus*, *massiliense*, and *bolletii*, respectively; MTB, MAV, MI, MG, and MKA are *M. tuberculosis*, *M. avium*, *M. intracellulare*, *M. gordonae*, and *M. kansasii*, respectively; NC, negative control.

To validate the amplification efficacy and specificity of two primer sets, amplifications of clinical samples were conducted, including 25 Mab strains and six clinically prevalent pathogenic mycobacterial strains. The amplification patterns were demonstrated as Fig. 7, and the outcomes were in full agreement with the anticipated results. The amplification products of Mab-1 F/R were 410, 410, and 2,599 bp for subspecies *abscessus*, *massiliense*, and *bolletii*, respectively, and those of Mab-2 F/R were 1,775, 386, and 386 bp, respectively. Without sequencing, the three Mab subspecies can be rapidly differentiated solely by the disparities in the lengths of these products. Moreover, no bands were detected for other common mycobacteria, which showed the high specificity of the primers in practical detection.

## DISCUSSION

In spite of the earlier taxonomic debates, Mab is now classified as a single species consisting of three subspecies. Our results, whether based on core genes or on ANI, also support this classification. Meanwhile, the differential genomic regions of three subspecies, such as the deletion of MASB_RS06435 in both subspecies *abscessus* and *massiliense*, and the insertion of MAB_RS17790-MAB_RS17795 in subspecies *abscessus* indicated that the likely evolutionary order is subspecies *bolletii*, *massiliense,* and *abscessus* in turn. The phylogenetic analysis of core genes also supported this inference.

The clear ANI boundaries between 97.7 and 98.2% that have been observed among the three subspecies indicated that, as a criterion commonly used for species classification (95% ANI, 70% DDH), ANI can also be applied to the classification of Mab subspecies (98%ANI, 79% DDH [17]). The subspecies classification accuracy determined by this ANI threshold matches that of the phylogenetic analysis of core genes. However, the ANI-based approach has significant advantages in terms of speed and ease of operation.

As the analysis was extended to cover all Mab genomes, the demarcation of the ANI boundary becomes less distinct. Although the ANI values between the genomes and the reference genome in the same subspecies are greater than 98%, the ANI values between these genomes may be lower than 98%, which reflects the genetic continuity and the intra-subspecies variations. Meanwhile, our results clearly demonstrated the impact of designating different genomes as reference genomes on the classification results. Therefore, we recommend adopting a unified set of reference genomes for species/subspecies classification to enhance the consistency and comparability among different bodies of research. The genomes of the type strains are preferred over any genome within the subspecies or the genome with the highest similarity to the tested genome in terms of 16S rRNA and other characteristics.

After revising the subspecies of the genomes based on ANI and core gene clustering, our results revealed that intact *erm*(41) genes are not present in all subspecies *abscessus* strains, and a small percentage of subspecies *massiliense* harbor complete *erm*(41) genes, although this proportion is notably low. This underscores the necessity to revise our knowledge about subspecies *abscessus* and *massiliense*. Shallom et al. found that two strains of subspecies *massiliense* contained intact *erm*(41) genes (39), but truncated *erm*(41) genes in subspecies *abscessus* have not been reported. Evidently, the integrity of the *erm*(41) gene cannot be used as a discriminative marker for these two subspecies, and it cannot be assumed that all strains of subspecies *massiliense* lack inducible resistance. Moreover, our results confirmed that the *rpoB* gene alone is insufficient for differentiating Mab subspecies, despite being previously thought to possess the power for both Mab species and subspecies (12, 40).

Finally, the Mab subspecies identification method we developed based on subspecies-specific genes identified through pangenome analysis converts genome-based ANI comparison into single-gene test with completely identical discrimination results and enhanced clinical applicability. Similar to all genetic identification markers, our novel markers retain the potential for horizontal transfer and recombination between subspecies. However, results from this study have shown that these two markers can accurately differentiate all genomes in the current data set, suggesting their relative stability throughout subspecies evolution. While future variation cannot be excluded, we thought it was necessary to conduct periodic monitoring and validation with newly submitted genomic data. Moreover, the advantage of our markers lies in the drastic minimization of back mutation likelihood, which could cause confusion in subspecies classification in contrast to *ropB* or other single-gene markers. The technical characteristics of PCR determine that it can achieve rapid, simple, and culture-independent direct detection of clinical samples. Our design facilitated the result interpretation owing to sequencing-free and significant differences (exceeding 1,000 base pairs) among the amplification products. Moreover, these subspecies-specific regions of Mab can also be used as targets for other detection technologies to increase its detection sensitivity. It is regrettable that only one strain of subspecies *bolletii* in our culture collection was available for amplification verification of actual strains. This is primarily due to the fact that this subspecies is far less prevalent in China than the other two subspecies. Notably, however, the amplification results of this strain were in complete accordance with those derived from simulated amplification using 130 genomes of subspecies *bolletii*. We further anticipate that the method established herein will undergo broader clinical application and validation in the future.

### Conclusions

Correct species/subspecies identification is not only crucial for precise diagnosis and treatment strategies but is also important for epidemiological analysis and antimicrobial resistance-related research. The accuracy in species identification of WGS has been widely recognized, and ANI has now become the gold standard (41, 42). In this study, the applicability of ANI for Mab subspecies identification has been verified, and the comprehensive analysis of all Mab genomes has revised the options of the three Mab subspecies and accurate genetic markers for subspecies identification.

## SUPPLEMENTAL MATERIAL

Figure S1 jcm.00697-25-s0001.pdf Re-evaluation of the subspecies.

Tables S1 and S2 jcm.00697-25-s0002.xlsx Reclassification of *Mycobacteroides abscessus* at the subspecies level.

Supplemental legend jcm.00697-25-s0003.docx Legend for Fig. S1.

ASM does not own the copyrights to Supplemental Material that may be linked to, or accessed through, an article. The authors have granted ASM a non-exclusive, world-wide license to publish the Supplemental Material files. Please contact the corresponding author directly for reuse.
