# Supplementary material for: Genomic-enabled classification of three Mycobacteroides abscessus subspecies and an effective subspecies-specific identification method
Source: J Clin Microbiol. 2025 Jul 24;63(8):e00697-25. doi: 10.1128/jcm.00697-25 (PMC12345259; doi:10.1128/jcm.00697-25)
Supplement: Figure S1 — Re-evaluation of the subspecies. [file jcm.00697-25-s0001.pdf]

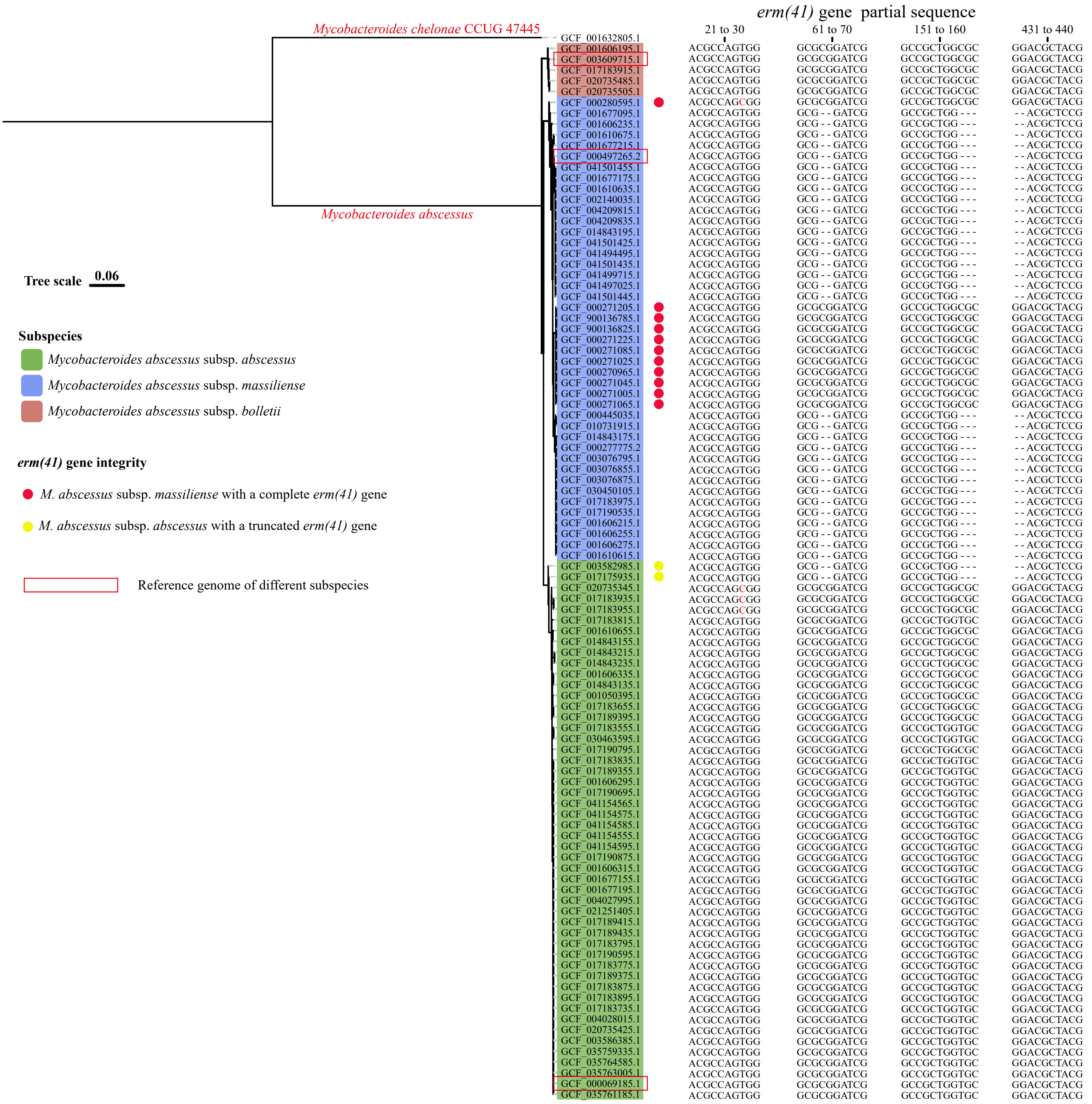

erm(41) gene partial sequence
21 to 30
61 to 70
151 to 160
431 to 440
Mycobacteroides chelonae CCUG 47445
Mycobacteroides abscessus
Tree scale 0.06
Subspecies
Mycobacteroides abscessus subsp. abscessus
Mycobacteroides abscessus subsp. massiliense
Mycobacteroides abscessus subsp. bolletii
erm(41) gene integrity
M. abscessus subsp. massiliense with a complete erm(41) gene
M. abscessus subsp. abscessus with a truncated erm(41) gene
Reference genome of different subspecies
