# Supplementary material for: Genomic-enabled classification of three Mycobacteroides abscessus subspecies and an effective subspecies-specific identification method
Source: J Clin Microbiol. 2025 Jul 24;63(8):e00697-25. doi: 10.1128/jcm.00697-25 (PMC12345259; doi:10.1128/jcm.00697-25)
Supplement: Supplemental legend — Legend for Fig. S1. [file jcm.00697-25-s0003.docx]

**Figure** **S1**. Re-evaluation the subspecies classification of the 13 genomes with abnormal *erm*(41) genes on the phylogenetic tree of core genes from 85 complete genomes. Red and yellow dots represent subspecies *massiliense* with full-length *erm*(41) gene and a truncated *erm(*41) gene, respectively. The right side shows the key mutation and deletion in the *erm*(41) gene sequence.
